# Optimization of a mouse model of pancreatic cancer to simulate the human phenotypes of metastasis and cachexia

**DOI:** 10.1186/s12885-024-12104-0

**Published:** 2024-04-04

**Authors:** Victoria Spadafora, Benjamin R. Pryce, Alexander Oles, Erin E. Talbert, Martin Romeo, Silvia Vaena, Stefano Berto, Michael C. Ostrowski, David J. Wang, Denis C. Guttridge

**Affiliations:** 1 Department of Pediatrics, Darby Children’s Research Institute, 416, Medical University of South Carolina, 173 Ashley Avenue, Charleston, SC 29425 USA; 2 Department of Health and Human Physiology, and the Holden Comprehensive Cancer Center, University of Iowa, Iowa, 52242 USA; 3 Hollings Cancer Center, Medical University of South Carolina, Charleston, SC 29425 USA; 4 Department of Neuroscience, Medical University of South Carolina, Charleston, SC 29425 USA; 5 Department of Biochemistry and Molecular Biology, Medical University of South Carolina, Charleston, SC 29425 USA

**Keywords:** Pancreatic ductal adenocarcinoma, Metastasis, Cachexia, Skeletal muscle

## Abstract

**Background:**

Pancreatic ductal adenocarcinoma (PDAC) presents with a high mortality rate. Two important features of PDAC contribute to this poor outcome. The first is metastasis which occurs in ~ 80% of PDAC patients. The second is cachexia, which compromises treatment tolerance for patients and reduces their quality of life. Although various mouse models of PDAC exist, recapitulating both metastatic and cachectic features have been challenging.

**Methods:**

Here, we optimize an orthotopic mouse model of PDAC by altering several conditions, including the subcloning of parental murine PDAC cells, implantation site, number of transplanted cells, and age of recipient mice. We perform spatial profiling to compare primary and metastatic immune microenvironments and RNA sequencing to gain insight into the mechanisms of muscle wasting in PDAC-induced cachexia, comparing non-metastatic to metastatic conditions.

**Results:**

These modifications extend the time course of the disease and concurrently increase the rate of metastasis to approximately 70%. Furthermore, reliable cachexia endpoints are achieved in both PDAC mice with and without metastases, which is reminiscent of patients. We also find that cachectic muscles from PDAC mice with metastasis exhibit a similar transcriptional profile to muscles derived from mice and patients without metastasis.

**Conclusion:**

Together, this model is likely to be advantageous in both advancing our understanding of the mechanism of PDAC cachexia, as well as in the evaluation of novel therapeutics.

**Supplementary Information:**

The online version contains supplementary material available at 10.1186/s12885-024-12104-0.

## Background

Pancreatic ductal adenocarcinoma (PDAC) is a devastating disease. PDAC currently ranks third among US cancer-related deaths but is predicted to rank second by 2030, exceeding the mortality rates of breast, colorectal, and prostate cancers [1, 2]. Advances have been made in abdominal imaging, surgical techniques, and chemotherapy regimens, all of which have led to an improvement in the number of patients surviving five years after their diagnosis from 4 to 12%. Nevertheless, the prognosis for this cancer remains extremely poor, with annual mortality rates approaching incidence rates, and survival being the lowest amongst all solid tumor malignancies [3]. Complete surgical resection continues to offer the only hope for long-term survival [4].

PDAC arises from a precursor lesion called Pancreatic Intraepithelial Neoplasia (PanIN) [5]. The development of these lesions and their transition from PanIN stages 1 to 3 to PDAC is characterized by a series of genetic alterations. These include activation of the *KRAS* oncogene that occurs in greater than 90% of PDAC cases, and the inactivation of tumor suppressor genes, such as *TP53*, *CDKN2A*, *SMAD4*, and *BRCA1/2* [5, 6]. The progression of PDAC is also influenced by a dynamic tumor microenvironment consisting of stromal immune cells and fibroblasts that promote a desmoplastic reaction and favor resistance to chemotherapy and other cytotoxic agents [7].

There are several factors that underlie a poor outcome in PDAC. The most severe is the high degree of metastatic dissemination to adjacent organs at disease presentation, with the most common site of metastasis being in liver [8]. Even in operable cases where the primary tumor has been completely resected, 75% of PDAC patients will succumb to a metastatic relapse in less than 5 years following their operation [9]. Another major factor contributing to lower survival is the cachexia syndrome, which is highly prevalent among PDAC patients, even at the initial stages of the disease [10]. Cachexia is characterized by involuntary weight loss mainly due to the depletion of adipose tissue and skeletal muscle mass. Estimates indicate that as many as 85% of PDAC patients lose on average 14% of their pre-illness weight [11, 12]. Data from our own PDAC patient registry showed that patients with weight loss > 10% had worse survival outcomes [13]. Additionally, cachectic patients are often weak, fatigued, and less tolerant to chemo- and radiotherapy, leading to a lower quality of life [14].

Various mouse models of PDAC have provided key insights into the etiology of this disease. The genetically engineered mouse model (GEMM), known as the KPC mouse (*Pdx1-Cre, LSL-Kras*^+*/G12D*^, LSL-*Trp53*^*R172H/*+^), was shown to faithfully recapitulate the genetics and pathology of PDAC [15]. In this model, the *Pdx1* promoter drives Cre recombinase to express the lox-stop-lox (LSL) mutant *Kras*^*G12D*^ gene, as well as the LSL mutant allele of *Trp53*. KPC mice develop pre-invasive PanIN and progress to advanced disease, exhibiting metastasis to the liver, lungs, peritoneum, and lymph nodes [15]. However, the KPC model is not without its limitations. KPC mice display a widely variable tumor latency (6–50 weeks) requiring sizable cohorts to be maintained, and their median survival is 150 days. This can make pre-clinical dosing studies time consuming, especially when including survival as an outcome measure [16, 17]. In addition, although KPC mice have been utilized as a model of cachexia [18–20], we find that due to the heterogeneity of the model, pancreas pathology does not consistently correlate with the cachexia phenotype as seen in PDAC patients [18]*.* Another widely used model to study PDAC is the orthotopic implantation of KPC cells into the pancreas of immunocompetent mice. In this model, varying numbers of KPC cells (5 × 10^4^—3 × 10^6^) are implanted into the tail of the pancreas [21, 22]. In all cases, this leads to rapid tumor development and a median survival between 14 – 35 days. Cachexia is observed, but metastases are not consistently reported before mice succumb to their disease [23–26]. In a similar setting, the KPP mouse that we designed as a GEMM of pancreatic cancer-induced cachexia (*Ptf1a*^+*/ER−Cre*^; *LSL-Kras*^+*/G12D*^; *Pten*^*f/f*^), develops pancreatic adenocarcinoma and progressive cachexia, but does not recapitulate a metastatic phenotype [18]. With the possibility of gaining new insights into the pathology of PDAC and performing pre-clinical studies with novel therapeutics, we set out to create a new versatile mouse model of PDAC, which could reliably recapitulate both human phenotypes of metastasis and cachexia.

## Methods and materials

### Established cell lines

KPC cell lines, KPC, KPC 2838c3, and KPC 6419c5, were previously utilized and generated as described [27, 28]. Cachexia cell lines Colon-26 (C-26) and Lewis Lung Carcinoma (LLC) were obtained from the National Cancer Institute as previously described [29]. All cell lines were cultured in DMEM with 10% FBS and 1% penicillin–streptomycin in a humidified incubator at 37^0^C with 5% CO_2_.

### Animals

All animals were housed at the animal facility at the Medical University of South Carolina (MUSC) with constant temperature and humidity and fed a standard diet ad libitum with free access to water. All mouse treatments were approved by the Institutional Animal Care and Use Committee (IACUC). For surgical procedures, all mice were anesthetized by isoflurane inhalation. Either a rodent-specific hair trimmer or chemical hair remover were utilized to remove animal hair. All C57BL/6J mice for surgical procedures and LLC xenografts were purchased from Jackson Laboratories. CD2F1 male mice were obtained from Charles River Laboratories. *Kras*^+/G12D^, *Ptf1a*^+/ER−Cre^, and *Pten*^f/f^ mouse strains were individually purchased from Jackson Laboratories and crossed to generate the KPP line.

### Tumor models

C-26 cells (1.0 x 10^6^ in 100 µl of PBS) were injected subcutaneously into the flank of CD2F1 male mice as previously described [29]. LLC cells (0.5 × 10^6^ in 50 µl of PBS) were injected into the gluteus muscles. Age-matched controls were injected with PBS. Tumor development in KPP mice was induced by intra peritoneal injections of tamoxifen at 1 mg/10 g body weight for 5 days consecutively as previously described [18]. All mice were observed after injection until reaching IACUC endpoint criteria and sacrificed for blood withdrawal via cardiac puncture, muscle isolation, and muscle weight measurements.

### Orthotopic model of PDAC

Surgical procedures for orthotopic PDAC were performed on C57BL/6 female or male mice as described previously [21] with slight modifications. In brief, murine pancreatic cancer cell lines suspended in ice cold PBS and Matrigel (in a ratio of 1:1) were loaded into a pre-chilled 1 mL syringe with a 27G pre-attached needle. A total of 40 µL of PBS/Matrigel cell mixture was injected per mouse. The pancreas of the mouse was accessed through a small, up to 1cm incision on the skin and body wall at the left flank of mice. The tail of the pancreas was lifted out of the body cavity with a pair of forceps and pancreatic cancer cell lines were injected. A clear water bubble was observed when a successful injection was achieved. The body walls of the mice were closed with 5–0 absorbable sutures (Z463G, Ethicon) and the skin of the mice was closed with 7mm metal clips. For deeper injections, proximal to the head of the pancreas, skin incisions up to 1.2 cm in length were made in the same location, at the mid-line of the left flank. After identifying the spleen, an incision was made on the body wall to the right side of the mid-point of the spleen. Then, the lower edge of the incised body wall was lifted with a pair of forceps and pulled slightly downward, in order to look for the tip of the pancreas located under the tip of the spleen. Another pair of forceps was utilized to lift the pancreas out of the body cavity until both the pancreas and spleen were exposed outside the peritoneum cavity. The injections were performed on or close to the bulky head of the pancreas at the level of the head of the spleen. After injection, the body wall and skin of the mice were closed in the same way as pancreas tail injections. When determined, tumor weights were weighed and recorded (Supplemental Table 1 and 2). All injected mice were observed up to 5 months post injection or until they reached early removal criteria (Supplemental Table 1 and 2).

### Generation of the KPCML1 cell line

A visible KPC liver metastatic nodule was excised in a sterilized animal manipulation hood and placed in ice cold DMEM + 10% FBS. The nodule was transferred in media into a tissue culture hood. After rinsing the tissue with PBS to remove excess blood, the nodule was minced into approximately 3.0 mm pieces and digested in an enzyme solution (0.05 mg/ml collagenase I, 0.05 mg/ml collagenase IV, Hyaluronidase 0.025 mg/ml, DNase I 0.01 mg/ml in HBSS) for 30 min at 37°C in a tissue culture incubator. The supernatant was transferred to a 50 ml conical tube containing full DMEM media and the remaining pieces were further digested for another 15 min. All digested supernatants were pooled together and passed through a 100 µM cell strainer (BD FALCON). After washing the strainer twice with DMEM + 10% FBS, cells were cultured in a 10 cm tissue culture dish until they reached 70 to 80% confluence. Cells were then harvested by trypsinization and split at 1:80 for subculturing. After 3 rounds of similar subculturing and when cells were observed to grow uniformly on tissue culture dishes, cells were expanded into multiple dishes and subsequently cryopreserved in DMEM + 10% FBS + 10% DMSO solution. The generation of this new cell line was referred to as KPCML1 (KPC Metastasis Large nodule 1).

### Cardiac and portal vein implantations of KPC cell lines

For cardiac injection of pancreatic cancer cell lines, mice under anesthesia were set in a supine position. A one milliliter syringe attached to a 27G needle was loaded with 100 µl of tumor cells at 10^4^ cells/ml PBS. Any tumor cells remaining on the outside of the needle were cleaned by wiping with a cotton ball soaked in 70% ethanol. The needle was inserted into the chest of the mice with the bevel side facing the left side of the mouse at a 20° angle under the sternum. When the needle reached the point half the distance between the front limb and the bottom of the sternum, the syringe was drawn to confirm the return of blood. Then, tumor cells were pushed into the left ventricle of mice. The needle was held in this position for 5 s to let the blood wash away the remaining cells and then it was withdrawn from the mouse. For portal vein injection, the syringe and tumor cell/PBS mixture were prepared the same as the heart puncture except that after loading tumor cell mixture into the syringe, the needle was switched from a 27G to a 30G. Mice under anesthesia were set at a supine position. A 2 cm incision was made on the skin and body wall on the left side of the abdomen between the sagittal and median plane below the edge of the ribs. A 4.0 cm × 4.0 cm gauze pad soaked with sterile 0.9% NaCl solution was set beside the incision on the left side of mice. A cotton swab dipped in 0.9% NaCl solution was utilized to push the intestine and colon out of the body cavity onto the damp gauze pad. The gauze was folded back over the organs to keep the intestine and colon moisturized. The cotton swab was utilized to push the remaining intestine and colon further left to expose the portal vein. The loaded 1.0 mL syringe with 30G needle was inserted into the portal vein at a 5° angle. After injection, the needle was held in position for 5 s to wash away any remaining tumor cells. Approximately 2 to 5 pieces of 0.3 to 0.4 cm hemostat gauze were set on the injection site. While pushing down with the cotton swab, the needle was pulled out of the portal vein and pressure was continuously applied for 5 min on the hemostat gauze to stop the bleeding. Then the hemostat gauze was removed, and the intestine and colon were pushed back into the abdominal cavity. The body wall was then closed by absorbable sutures and the skin closed by metal clips. After these procedures, mice were observed in the same way as the other tumor models for up to 5 months.

### Cell growth curves

KPC and KPCML1 cell lines were plated at 2,500 cells per well in a 24 well plate in triplicate. Cells were trypsinized and counted every 24 h for 5 days. Phase contrast images were obtained daily to monitor morphological differences between the cell lines.

### Determination of sex in the KPC cell line

DNA was isolated from the KPC cell line using the Qiagen DNeasy Blood and Tissue Kit per manufacturers protocol. DNA was isolated from male and female mouse tails using 200 µl of tail lysis buffer (10 mM Tris–HCl pH 8.3, 50 mM KCl, 1 mM MgCl2, 0.45% NP-40, 0.45% Tween-20) and 0.2mg/mL Proteinase K. The tails were digested overnight at 55°C in a hybridization oven while rotating. The next day, Proteinase K was heat inactivated at 85°C for 1 h. Both cell and mouse DNA were tested for the presence of male specific gene *sry* using forward primer: 5’-TGGGACTGGTGACAATTGTC-3’ and reverse primer: 5’-GAGTACAGGTGTGCAGCTCT-3’. The 20 µl PCR reaction containing 1 × PCR buffer (Qiagen), 0.2 mM each of dNTP, 1 M betaine, 0.5 µM each of forward and reverse primers, 0.1 µl Taq DNA polymerase and 1 µl of sample DNA was incubated on the thermocycler using the following protocol: 94°C for 3 min, 35 cycles x (94°C for 30 s, 60°C for 30 s, 72°C for 1 min), 72°C for 3 min and 4°C hold.

### Histopathology and immunohistochemistry

Pancreas tumors and visible metastasis nodules on the liver were fixed in formalin overnight and switched to 70% ethanol. Livers and pancreases in ethanol were processed by the Translational Science Core at the Hollings Cancer Center for paraffin-embedding, sectioning, and hematoxylin–eosin staining. The same metastatic lesions were further characterized by immunohistochemistry staining with α-smooth muscle actin and cytokeratin 19. Slides were observed and representative images were acquired with a Zeiss microscope.

### Digital Spatial Profiling (DSP)

Formalin fixed and paraffin embedded pancreas tumors and their corresponding metastatic liver lesion were sectioned and then analyzed with the Nanostring GeoMx DSP at the MUSC Translational Science Laboratory. Slides were processed as per the GeoMx DSP slide prep protocol. In brief, deparaffinized slides were subjected to antigen retrieval with DAKO antigen retrieval solution and blocked 1 h in Buffer W. Slides were hybridized with oligonucleotide labeled antibody probes from the Immune Cell Profiling Panel Mouse Protein Core and fluorescently stained with three morphology markers targeting macrophages (F4/80), total immune cells (CD45), and tumor bed (pan cytokeratin CK) as well as a nuclear stain (SYTO 13). Regions of interest (ROI) were circled based on areas positive for all three of these morphology markers. Oligos encircled in each ROI were cleaved and released after being targeted by UV light. The oligos were collected in a 96 well plate and hybridized to barcodes before being counted on the nCounter platform. Quality control, normalization and heat map generation were all performed using the GeoMx software as recommended from Nanostring.

### Cachexia endpoint measurements

The body weight of mice at end point of observation was calculated as BW = (TMBW – TW – AW) + BlW. In this equation, BW is body weight, TMBW is total measured body weight, TW is tumor weight, AW is ascites weight and BlW is the weight of the blood withdrawn. Hindlimb muscles (tibialis anterior, gastrocnemius, and quadriceps) were weighed and compared to the same muscles of age matched controls that had been orthotopically injected with Matrigel. If adipose tissue was found, it was also weighed and compared to age matched controls. Spleens were dissected out of the surrounding tumor and the heart removed from the thoracic cavity and weighed.

### Serum ceramides

Mice were anesthetized using vaporized isoflurane. Approximately 1ml of blood was drawn from the mice by inserting a 27-gauge needle into the left ventricle of the heart, as previously described for tumor cell cardiac injection. Blood was allowed to clot at room temperature for approximately 25 to 30min and then spun down at 5,000 rpm for 10 min at 4°C. The supernatant (serum) was transferred to another tube and frozen at -80°C until use. For lipidomic analysis, 100 µl of serum from tumor bearing mice and their age matched controls were sent to the MUSC Lipidomics Shared Resource for a general ceramide and sphingolipid panel. Separations for sphingolipids are performed by HPLC–MS/MS analyses at the MUSC Lipidomics Shared Resource. The equipment consisted of a Thermo Scientific Vanquish uHPLC system coupled to a Thermo Scientific Quantum Access Max triple quadrupole mass spectrometer equipped with an ESI probe operating in the multiple reaction monitoring (MRM) positive ion mode. Chromatographic separations are obtained under a gradient elution on a C8 column using a mobile phase with ammonium formate, formic acid in water and methanol, as previously described. Prior to analysis samples undergo an ethyl acetate/isopropanol liquid–liquid extraction. Quantitative analyses of sphingolipids are based on eight-point calibration curves generated for each target analyte. The synthetic standards along with a set of internal standards are spiked into an artificial matrix; they are then subjected to an identical extraction procedure as the biological samples. These extracted standards are then analyzed with the samples by the HPLC–MS/MS. Peaks for the target analytes and internal standards are recorded and processed using the instrument’s software. Plotting the analyte/internal standard peak area ratios against analyte concentrations generates the sphingolipid specific calibration curves. Any sphingolipids for which no standards are available are quantitated using the calibration curve of its closest counterpart [30–32].

### Mouse and human RNA-seq analysis

RNA-seq analysis of pancreatic cancer models was completed as previously described with slight modifications [18]. In brief, total RNA was isolated using Trizol reagent (Life Technologies, CA) as recommended by the manufacturer from snap-frozen tibialis anterior muscles. RNA quality was assessed using an Agilent 4200 TapeStation and RIN^e^ values ranged from 8–10. Total RNA (250 ng) was utilized to construct cDNA libraries with the New England Biolabs NEBNext® Poly(A) mRNA Magnetic Isolation Module (Cat# 7490L) and Ultra II Directional RNA Library Prep Kit from Illumina (Cat# 7760L) as recommended by the manufacturer. Dual-indexed libraries were pooled and sequenced at VANTAGE (Vanderbilt University Medical Center) on an Illumina NovaSeq 6000 to a depth of approximately 25 million paired-end 150 bp reads per library. Raw sequencing files for expression changes in muscle biopsies of male cachectic pancreatic cancer patients or control patients were retrieved through the ﻿Gene Expression Omnibus (GEO, RRID:SCR_005012) and analyzed for differential expression analysis (GEO GSE13352) [20]. Sequencing datasets for expression changes in muscles from control and cachectic mice are available from GEO, with the following accession link, https://www.ncbi.nlm.nih.gov/geo/query/acc.cgi?acc=GSE251864, using the reviewer code, edulcucilvchpit. Analysis was performed in R. Briefly, transcript quantification of raw FASTQ sample sequencing files was performed with Salmon k-mer counting against an index derived from the transcript FASTA file of mouse genome assembly mm10 or human genome assembly hg38 (UCSC) [33]. Use of Salmon was achieved in R through Herper to establish a miniconda environment. DESeq2 (RRID:SCR_015687) was used for differential expression analysis between control replicates and cancer condition replicates [34]. Differentially accessible peaks of genes were filtered for a fold change > 50% and a false discovery rate (FDR) of < 0.05 with the Benjamini–Hochberg correction. The package clusterProfiler was used to perform gene ontology enrichment for biological processes for each differential expression analysis [35].

### Statistical analysis

Tumor-bearing mice comparisons to their respective controls used two tailed t tests with unequal variance in Microsoft Excel to determine significance. Statistical significance was determined as *p* < *0.05*. One-way ANOVA was performed to compare 6-month-old mice with and without metastasis to their Matrigel controls with a Dunnett’s multiple comparisons test in Graph Pad Prism. Statistical significance was determined as adjusted *p-value* < *0.05*.

## Results

### Orthotopic implantation of KPC cells into the tail or head of the pancreas is not sufficient to consistently recapitulate an advanced form of human PDAC

We initiated our study by using a KPC cell line generated from a *Pdx*^*Cre*^*; Kras*^+*/G12D*^*; Trp53*^*fl/fl*^ GEMM [27, 28] that we refer to as KPC. This line was used in our previous study to investigate the role of NF-_K_B in regulating PDAC development [21]. Consistent with our previous protocol [21], we implanted 50,000 cells into the tail of the pancreas of 10-week-old female immunocompetent syngeneic mice. This led to 100% formation of primary tumors, but only 28% of tumor bearing mice developed metastatic liver nodules (Table 1). Transplantation of two other KPC lines, 2838c3 and 6419c5, derived from GEMMs (*Pdx*^*Cre*^*; Kras*^+*/G12D*^*; Trp53*^+*/R172H*^*; Rosa*^*YFP/YFP*^) that had been previously used to examine immune heterogeneity in the PDAC tumor microenvironment [36] led to similar high rates of primary tumor formation, but low rates of liver metastasis (Table 1).

**Table 1:** Orthotopic implantation of KPC cell lines

Sex	# of Mice	Cell Line	Cell #	# of Mice with Tumors	% Tumors	# of Metastases	% Metastasis
Orthotopic Implantations into the Tail of the Pancreas							
Female	7	KPC	50,000	7	100	2	28.6
Female	6	KPC 2838c3	50,000	6	100	0	0
Female	6	KPC 6419c5	50,000	6	100	0	0
Orthotopic Implantations into the Head of the Pancreas							
Female	7	KPC	10,000	6	85.7	1	16.7
Female	5	KPC 2838c3	10,000	5	100	0	0
Female	5	KPC 6419c5	10,000	5	100	0	0
Orthotopic Implantations into the Head of the Pancreas							
Male	6	KPC	5,000	6	100	0	0
Male	6	KPC	10,000	6	100	1	17
Male	5	KPC	20,000	5	100	2	40

Given that only a few mice developed metastasis characteristic of advanced disease in these initial experiments, we decided to modify our transplantation protocol. First, since human PDAC tumors are predominantly located in the head of the pancreas [37, 38], we mimicked this condition in recipient mice, reasoning that this would form tumors closer to the portal vein system and facilitate dissemination to the liver. Second, we decreased the number of injected cells, expecting that a lower implantation number would increase the latency of PDAC development, and in turn provide tumor cells more time to disseminate and form metastatic nodules. Surprisingly, although these modifications maintained high rates of primary tumor formation, they did not increase rates of metastasis above the previous transplantation conditions (Table 1). Thus, at least in recipient female mice, orthotopically implanting a relatively low number of KPC cells into the tail or head of the pancreas is not sufficient to consistently recapitulate the advanced form of human PDAC. To determine whether we could increase this efficiency by utilizing male mice, we orthotopically implanted KPC cells into the head of the pancreas, and at the same time reduced cell numbers from 20,000 to 10,000 and 5,000. Results showed that all three cohorts formed tumors with 100% efficiency, but only mice injected with 20,000 cells produced liver metastases, albeit still at a relatively low rate of 40% (Table 1).

### Orthotopic implantation from re-established KPC cells produces a high rate of metastasis

**Fig. 1:**
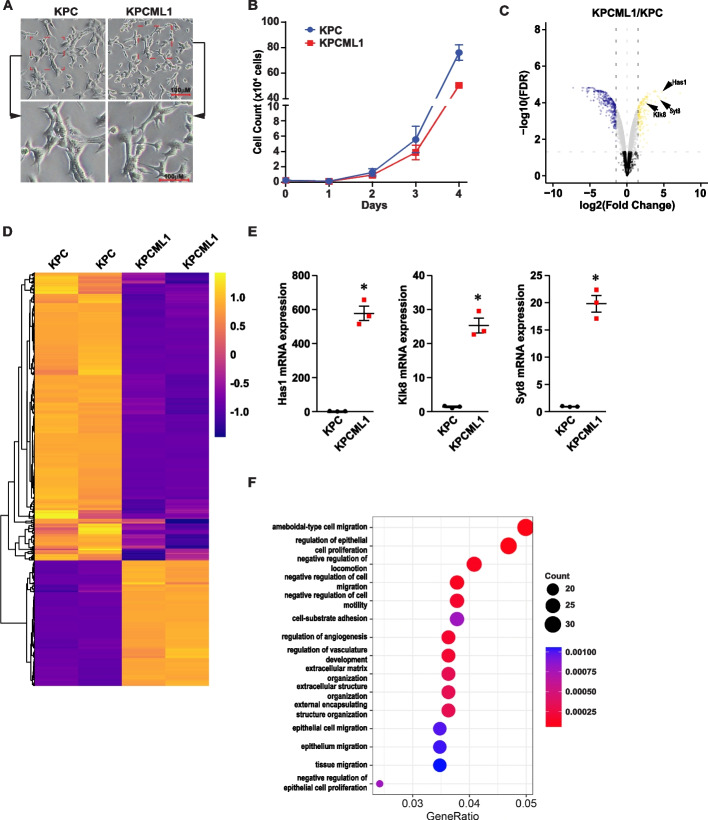
*Generation and characterization of the KPCML1 cell line*. **A** Representative phase contrast microscopic images of the KPC parental and KPCML1 metastatic cell lines, shown at two magnifications. **B** Graph depicting the growth of KPC and KPCML1 cell lines in culture over 4 days. **C** Volcano plot of DEGs from RNA-seq results comparing KPCML1 to KPC cells. Expression of *Has1*, *Syt8*, and *Klk8* genes are indicated by arrows. **D** Heatmaps of significantly altered genes (*p* < 0.05) expressed from KPCML1 (*n* = 2) versus KPC cells. **E** Quantitative real-time PCR analyses of *Has1*, *Syt8*, and *Klk8* genes in KPCML1 cells compared to KPC cells (*n* = 3). Data are shown as mean ± SEM. **F** Gene ontology analysis on differentially expressed genes from RNA-seq analysis of KPCML1 to KPC cells. * represents *p* < 0.0005, as determined by two tailed t tests with unequal variance

Given that strategies described above did not significantly influence the metastatic potential of transplanted KPC cells, we reasoned that the tumor cells that had successfully formed a metastatic lesion might have undergone phenotypic changes that enabled them to disseminate throughout the circulation and successfully establish a new niche in the liver. If such alterations did exist in cells contained within the metastatic lesion, a cell line derived from this lesion and implanted into the pancreas could potentially be more successful at generating consistent liver metastases. Thus, we isolated one of the metastatic liver nodules and subcultured the resulting cells over several passages to re-establish a KPC line that we named, KPCML1 (Fig. S1). The morphology and growth characteristics of KPCML1 cells did not significantly differ from the parental line (Fig. 1A and B). However, when comparing differentially expressed genes (DEGs) by RNA sequencing (RNA-seq), we found that KPCML1 cells contained 272 genes that were upregulated and 507 genes that were downregulated compared to KPC cells (Fig. 1C and D; FDR of < 0.05 and a fold cutoff of 1.5-fold). Three of the highest upregulated DEGs from KPCML1 cells, *Hyaluronan Synthase 1* (*Has1*), S*ynaptotogmin 8* (*Syt8*), and *Kallikrein-8* (*Klk8*), have been associated with the progression of PDAC [39–41]. The upregulation of these three genes were further confirmed by qRT-PCR (Fig. 1C and E). Furthermore, Gene Ontology (GO) analysis of upregulated transcripts from KPCML1 cells revealed that the most significant biological processes were related to cell proliferation, migration, adhesion, angiogenesis, and extracellular matrix organization, all of which are recognized properties of metastasis (Fig. 1F).

**Fig. 2:**
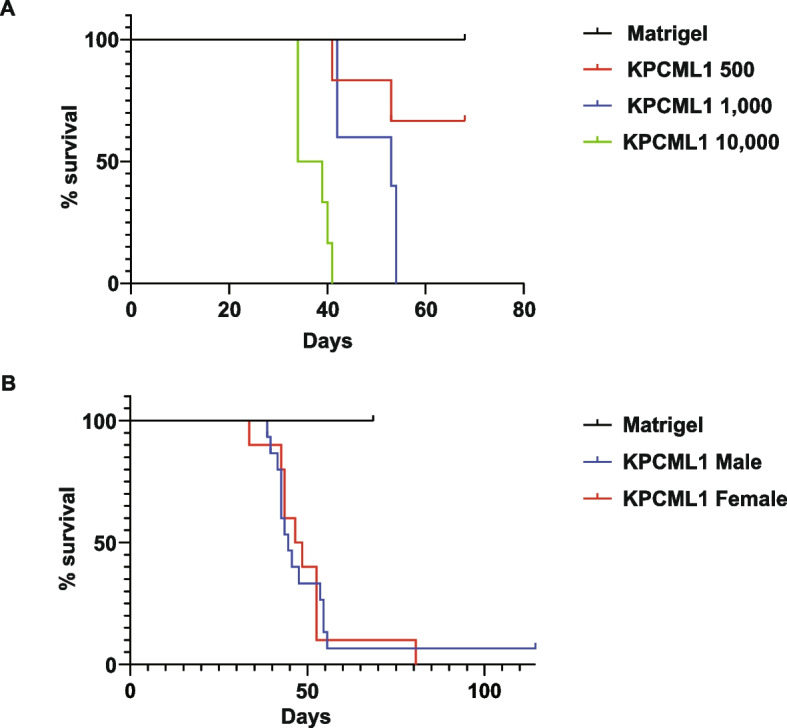
*KPCML1 mice exhibit an extended survival which is not sexually dimorphic*. **A** Survival graphs of mice implanted with either Matrigel control or 10,000, 1,000, and 500 KPCML1 cells in the head of the pancreas. **B** Survival graphs of male and female recipient mice implanted with Matrigel or 1,000 KPCML1 cells in the head of the pancreas, as determined by two tailed t tests with unequal variance

Based on the phenotype of KPCML1 cells, we performed orthotopic injections into the head of the pancreas of male mice, again reducing the number of cells. Results showed that injections of 10,000 and 1,000 KPCML1 cells formed pancreatic tumors with 100% efficiency, and impressively, those mice implanted with 1,000 cells developed liver metastases at a rate of 80% (Table 2). Further, these mice had a median survival of 53 days compared to mice injected with 10,000 cells that survived on average to 37 days (Fig. 2A). In comparison, orthotopic injections performed with a much lower number, such as 500 cells, developed primary tumors in only half the number of mice. Similarly, the rate of tumor formation was low using 200 KPCML1 cells, and with these transplants only 1 metastatic liver lesion was observed, even after following these mice for over 6 months (data not shown). Thus, orthotopic implantation of 1,000 KPCML1 cells appeared to be an optimal experimental condition to phenocopy the metastatic state commonly observed in PDAC patients.

**Table 2:** Orthotopic implantation of KPCML1 cells

Sex	# of Mice	Cell Line	Cell #	# of Mice with Tumors	% Tumors	# of Metastases	% Metastasis
Male	5	Matrigel	0	0	0	0	0
Male	6	KPCML1	500	3	50	2	67
Male	5	KPCML1	1,000	5	100	4	80
Male	6	KPCML1	10,000	6	100	3	50

Because our earlier experiments involving female mice were limited to orthotopic implantations of only the parental KPC cell lines, we wanted to investigate whether this increased metastatic rate with the KPCML1 cell line was sexually dimorphic. To do this, implantations were repeated with KPCML1 cells into the pancreas of both male and female mice. Although genotyping for sex determined that KPCML1 cells are of male origin (Fig. S2), transplantation results showed equal rates of tumor formation, liver metastasis, and survival between male and female mice (Fig. 2B and Table 3), indicating that PDAC in this orthotopic model is independent of sex.

**Table 3:** Orthotopic implantation of KPCML1 cells

Sex	# of Mice	Cell Line	Cell #	# of Mice with Tumors	% Tumors	# of Metastases	% Metastasis
Male	10	KPCML1	1,000	10	100	4	40
Female	10	KPCML1	1,000	10	100	3	30

**Fig. 3:**
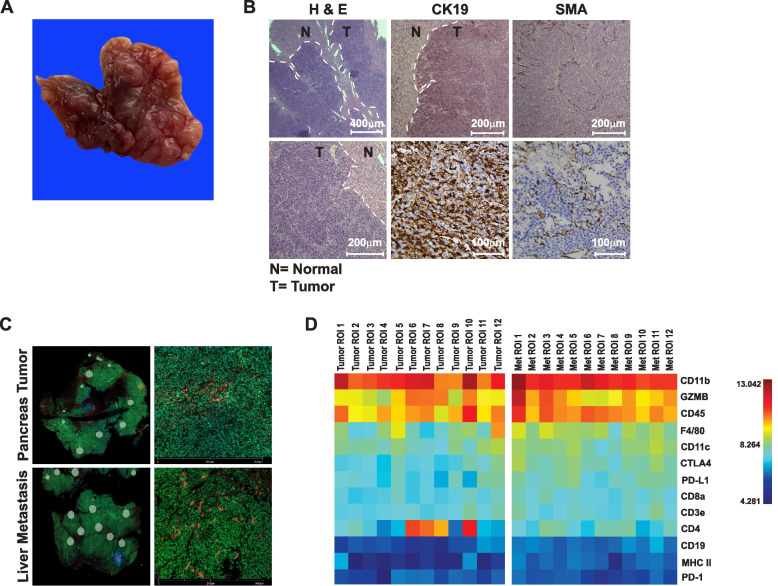
*The tumor immune microenvironments of KPCML1 primary and metastatic lesions are conserved.* **A** Representative photo of an excised liver with KPCML1 metastatic nodules. **B** Representative images of metastatic tumors lesions (T) and adjacent normal liver tissue (N; where indicated) stained by H&E, CK19, and SMA. **C** Digital spatial profiling of an immune-oncology panel comparing the primary and metastatic KPCML1 tumors; images on the left depict the selected regions of interest (ROIs) and on the right is a representative image of an ROI from the primary and metastatic KPCML1 tumor at higher magnification. **D** A cluster of immune markers were identified from the 12 selected ROIs and heat map profiles were generated and compared between the primary and metastatic tumor

Metastatic nodules from these mice varied in appearance and size but similar histological trends were observed throughout the liver (Figs. 3A and S3A). These nodules were positive in staining for CK19 and αSMA, confirming the presence of epithelial tumor cells and surrounding stroma, respectively (Fig. 3B). Due to reports that indicate that the driver mutations between the primary and metastatic lesions in PDAC are highly uniform [42], we sought to determine whether similar uniformity existed in the tumor microenvironment between primary and metastatic lesions. Two pairs of primary and metastatic lesions were compared by multiplex proteomics using a digital spatial profiling immuno-oncology platform. Analysis of 12 selected regions of interest (ROIs) revealed a remarkably similar immune cell profile between both the primary and metastatic tumor (Fig. 3C and D, and Fig. S3B and C), indicating that analogous to the genomic landscape [42], the tumor immune environment was also mainly conserved.

Since the blood supply is richer at the head of the pancreas, and blood vessels are thicker due to their close proximity to the superior mesenteric vein and portal vein, it was possible that the metastatic lesions we had observed in the liver could have resulted from an unintentional injection of KPCML1 cells directly into the blood stream. We thought our experimental design accounted for this by mixing pancreatic cancer cells with Matrigel, which solidifies and clogs blood vessels if cells are erroneously injected into the blood rather than into the pancreas. Nevertheless, to verify that metastatic lesions occurred from a *bona fide* dissemination of KPCML1 cells from the pancreas, we utilized a standard model of metastasis. In this model, tumor cells (1 × 10^5^—3 × 10^6^) are directly injected into the left ventricle of the heart, which pumps these cells into the systemic circulation allowing them to widely distribute and form metastases throughout the body [43, 44]. To adopt this model under our experimental conditions, we implanted either 1,000 KPC parental or KPCML1 cells into the heart of recipient mice. From these implantations, no metastatic nodules were detected in either the liver, lungs, or brain after 5 months (Table 4). We then tested the possibility that a more local release of tumor cells into the circulation had occurred with our injections. The venous system in the head of the pancreas directly drains into the superior mesenteric vein, which then drains into the portal vein leading to the liver. Thus, we repeated injections with KPC or KPCML1 cells directly into the portal vein of mice [45]. Significantly, even under these conditions, no liver, lung, or brain nodules were observed (Table 4). These observations support the conclusion that liver metastases resulted from the dissemination of circulating tumor cells originating from the primary tumor at the head of the pancreas after implantation of KPCML1 cells.

**Table 4:** Verification of metastasis from vascular implantation of KPC cell lines

Sex	# of Mice	Cell Line	Cell #	# of Mice with Tumors	% Tumors	# of Liver Nodules	# of Lung Nodules	# of Brain Nodules
Transplantation into the Left Ventricle Via Cardiac Puncture								
Male	6	KPC	1,000	0	0	0	0	0
Male	6	KPCML1	1,000	0	0	0	0	0
Transplantation into the Liver Via Portal Vein								
Male	5	KPC	1,000	0	0	0	0	0
Male	5	KPCML1	1,000	0	0	0	0	0

### Orthotopic implantation of KPCML1 cells promotes common cachexia endpoints

**Fig. 4:**
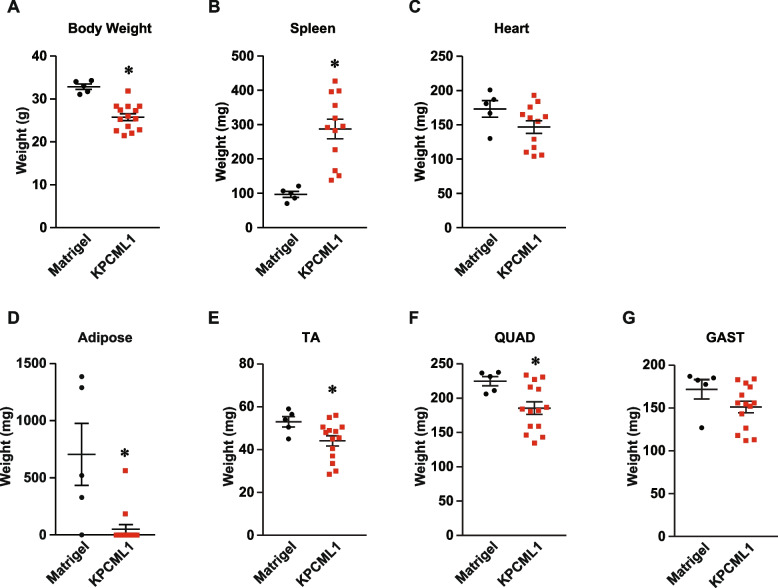
*KPCML1 mice recapitulate a cachexia phenotype.* Mice were transplanted with Matrigel as control or KPCML1 cells and standard endpoint measurements were obtained including body weight (**A**), spleen weight (**B**), heart weight (**C**), adipose weight (**D**), and weights from hindlimb muscles, tibialis anterior (TA) (**E**), quadriceps (QUAD) (**F**), and gastrocnemius (GAST) (**G**). Statistical analysis was performed using unpaired two-tailed t test with unequal variance. Data are shown as mean ± SEM. * represents *p* < 0.05

Having optimized conditions in an orthotopic mouse model of PDAC that exhibited efficient metastasis, we next sought to determine whether this same model could also recapitulate a cachexia phenotype. Injection of 1,000 KPCML1 cells into the head of the pancreas of male mice, compared to Matrigel control, led to changes in the most common cachexia endpoints, of which many were significant. We observed a reduction in total body weight, an enlarged spleen, an almost complete loss in gonadal fat, a significant reduction in the mass of tibialis anterior (TA) and quadriceps (QUAD) skeletal muscles, and a trend towards a loss of heart mass and gastrocnemius (GAST) muscle mass (Fig. 4A-G).

**Fig. 5:**
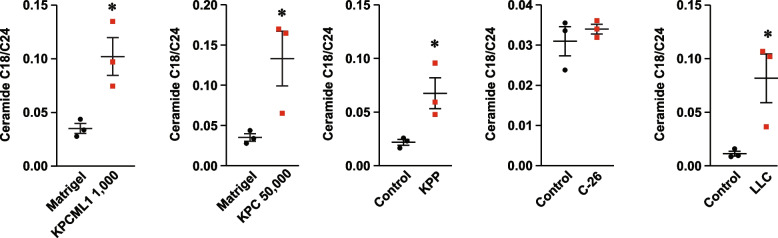
*The ceramide C18:C24 ratio is elevated in the serum of the KPCML1 orthotopic model and other mouse models of cancer cachexia.* The ceramide ratios of C18:C24 are shown from sphingolipid analyses performed with serum from KPCML1 mice, compared to the KPC orthotopic model and KPP, C-26, and LLC mouse models of cancer cachexia. Statistical analysis was performed using unpaired two-tailed t test. Data are shown as mean ± SEM. * represents *p* < 0.05

Recent studies from our laboratory have shown that circulating levels of sphingolipids are dysregulated in PDAC patients with cachexia. Specifically, we found that the plasma ceramide ratio of C18 to C24 (C18:C24) is elevated in cachectic PDAC patients compared to weight stable PDAC and control non-cancer patients [46]. This identified the C18:C24 ratio as a potential biomarker for PDAC-induced cachexia. To determine if a similar biomarker was relevant in our optimized orthotopic model of PDAC, we collected blood at endpoint from mice implanted with KPCML1 cells and performed a targeted profile of serum sphingolipids. Compared to our previously described general reduction of circulating sphingolipids from cachectic PDAC patients [46], we did not observe a similar dysregulation of circulating sphingolipids from mice implanted with 1,000 KPCML1 cells or 50,000 KPC cells, or from the KPP, Lewis Lung Carcinoma (LLC), and Colon-26 (C-26) mouse models of cancer cachexia (Fig. S4). However, akin to the elevation of the C18:C24 ratio in plasma from cachectic PDAC patients [46], this ceramide ratio was also significantly increased in our optimized orthotopic model of PDAC (Fig. 5). A similar significant increase in the C18:C24 ratio was observed with serum from mice orthotopically implanted with 50,000 KPC cells, serum from KPP and LLC models of cancer cachexia, and to a lesser degree with serum from the C-26 cachexia model (Fig. 5). These results imply that the C18:C24 ceramide ratio is independent of a specific mouse model, and instead may reflect its utility as a general biomarker of cancer cachexia.

### Metastasis, but not cachexia, is age dependent in KPCML1 orthotopic mice

Next, we addressed a discrepancy in the data presented in Tables 2 and 3, where implantation of 1,000 KPCML1 cells in the pancreas of male mice had resulted in rates of 80% and 40% metastasis, respectively. We realized that this may be due to the fact that the data reported in Table 2 were obtained from transplantation experiments performed with mice greater than 4-months of age, compared to mice in Table 3 and the rest of our study which were performed with younger mice between 10–12 weeks of age. Given that aging affects the tumor microenvironment to stimulate tumor initiation, progression, and metastasis [37, 38], this raised the question whether metastasis in our model was dependent on age. To test this, we orthotopically implanted KPCML1 cells in mice either 10-weeks (young) or 6-months (adult) of age. Results showed that both young and adult mice formed primary tumors with 100% efficiency (Table 5). However, implantation of KPCML1 cells in young mice led to metastases at a rate of 40%, whereas the rate in adult mice was 70%. These results are consistent with our earlier findings (Tables 2 and 3) and support that this orthotopic model of PDAC is age dependent. The metastatic liver lesions and primary tumors in adult mice were also analyzed using the same digital spatial profiling platform we had performed in young mice. Interestingly, here the immune environment in the metastatic lesion displayed an increase in infiltrating CD45^+^ cells that coincided with increases in CD11b marked myeloid cells, F4/80 marked macrophages, and CD11c marked dendritic cells (Fig. S5A-D). These markers revealed that unlike young PDAC mice where the immune tumor microenvironment is conserved, in the adult, the environment is less maintained and displays enhanced inflammation in the metastatic lesion.

**Table 5:** Orthotopic implantation of KPCML1 cells in young and adult mice

Sex	Age	# of Mice	Cell Line	Cell #	# of Mice with Tumors	% of Tumors	# of Metastases	% Metastasis
Male	Young	10	KPCML1	1,000	10	100	4	40
Male	Adult	10	KPCML1	1,000	10	100	7	70

**Fig. 6:**
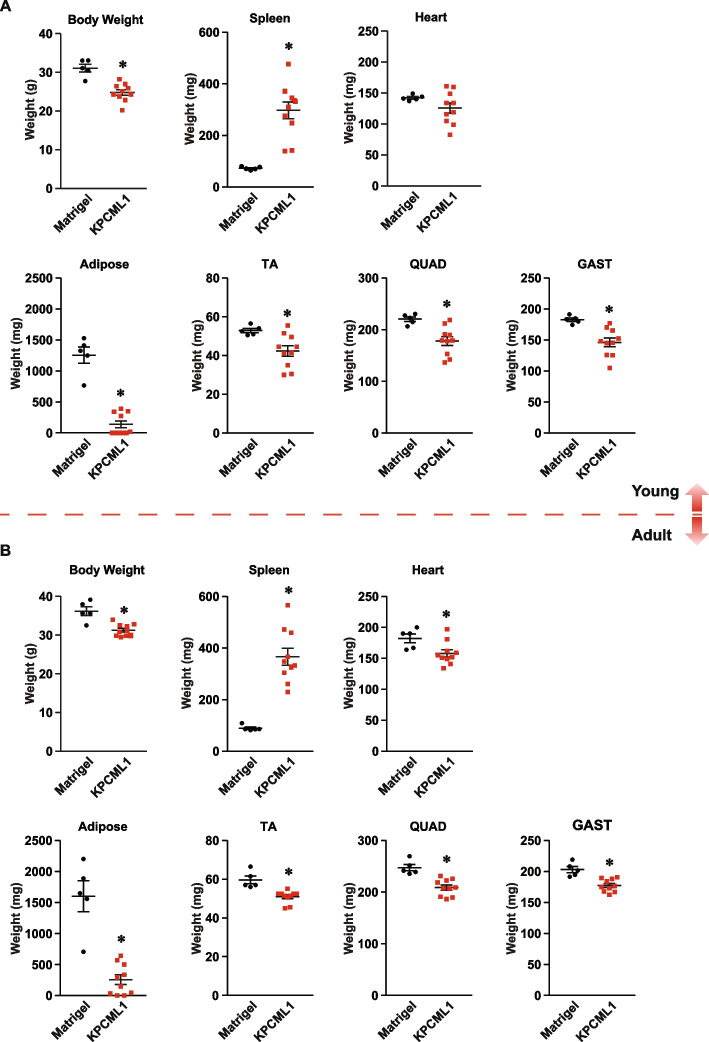
*The cachexia phenotype is recapitulated in both young and adult KPCML1 mice.* Young (**A**) or adult (**B**) mice were transplanted with Matrigel as control or KPCML1 cells and standard endpoint measurements were obtained including body weight, spleen weight, heart weight, adipose weight, and weights from hindlimb muscles, tibialis anterior (TA), quadriceps (QUAD), and gastrocnemius (GAST). Statistical analysis was performed using unpaired two-tailed t test with unequal variance. Data are shown as mean ± SEM. * represents* p* < 0.05

**Fig. 7:**
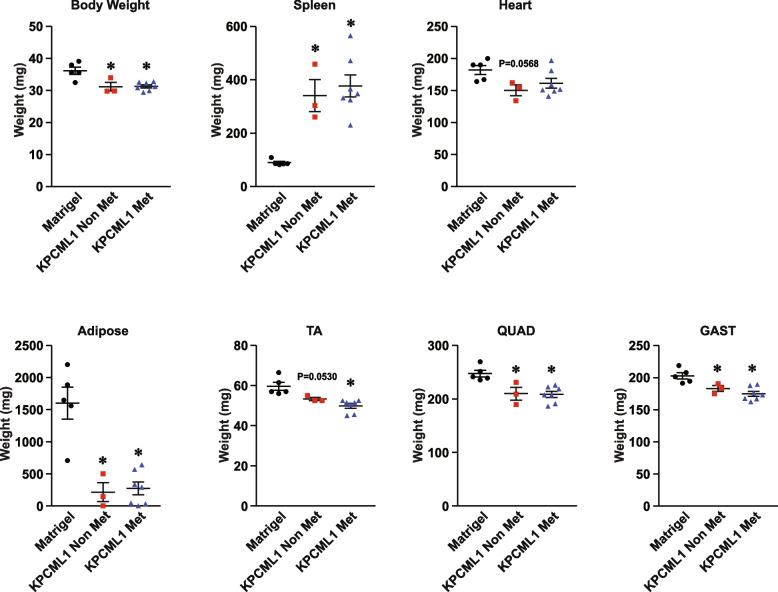
*Cachexia in KPCML1 mice occurs independently from metastasis*. Adult mice were transplanted with Matrigel control or KPCML1 cells and standard endpoint measurements were obtained from mice presenting with or without liver metastasis, including body weight, spleen weight, heart weight, adipose weight, and weights from hindlimb muscles, tibialis anterior (TA), quadriceps (QUAD), and gastrocnemius (GAST). Met, metastatic; Non Met, non metastatic, Statistical analysis was performed using 1-way ANOVA (*P* < 0.05) with Dunnett’s multiple comparisons test in Graph Pad Prism. Data are shown as mean ± SEM. * represents *p* < 0.05

We then asked if the cachexia phenotype is also dependent on age in KPCML1 mice. Results showed that regardless of age, the majority of the common cachexia markers including body weight, adipose mass and hindlimb muscle mass (TA, QUAD, and GAST) were significantly reduced in young and adult mice, with perhaps only slightly greater wasting observed in the adult setting (Fig. 6A and B). In addition, when we stratified the data from the adult cohort, between those that were metastatic and non-metastatic, we observed that body weight, adipose and muscle mass were significantly reduced in both conditions, with again only slightly greater wasting seen in the metastatic setting (Fig. 7). Together, these results suggest that cachexia in the KPCML1 orthotopic model occurs independently from metastasis, a finding that accurately phenocopies PDAC patients who commonly present with cachexia regardless of the stage of their disease [13].

### Transcriptomics reveal common pathways of muscle atrophy between KPCML1 metastatic and non-metastatic mice

**Fig. 8:**
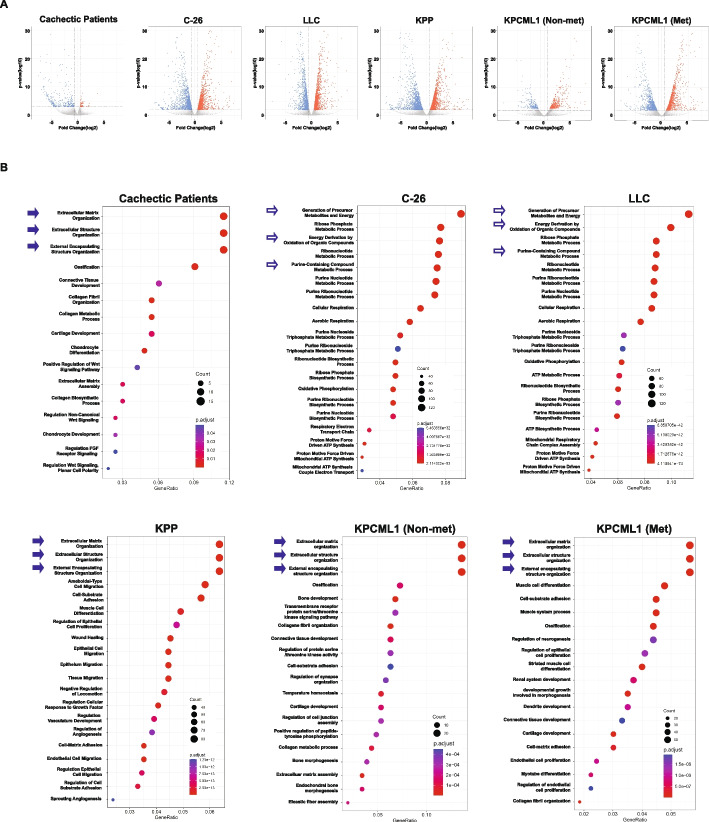
*KPCML1 mice exhibit a similar transcriptomic profile to that of cachectic PDAC patients.* **A** Volcano plots of DEGs from RNA-seq results from muscles of cachectic PDAC patients in comparison to muscles from KPCML1 mice (with and without metastasis) and KPP, C-26, and LLC mouse models of cancer cachexia. **B** GO graphs generated from the downregulated genes of subgroups shown in (**A**). Solid Arrows indicate pathways that are similar between muscles from cachectic pancreatic cancer patients and KPP and KPCML1 mouse models, in contrast to those pathways indicated by empty arrows that differ between muscles patients and C-26 and LLC mouse models

In the final analysis of our study, we performed RNA-seq to assess whether the KPCML1 model could recapitulate the cachexia phenotype of human PDAC by comparing transcriptomic profiles, as we had previously observed for KPP mice [18]. To analyze total DEGs, we used an FDR of < 0.05 and a fold cutoff of 1.5-fold. An independent analysis of our human data set revealed relatively few DEGs in muscles from cachectic PDAC patients versus weight-stable control patients (Fig. 8A), which was consistent with our original findings [18]. Significantly, the vast majority of DEGs were decreased in cachectic patients (315 out of 369 genes) (Table 6). This was in sharp contrast to muscles from the KPCML1 orthotopic model, where muscle DEGs were substantially greater in number (2019) and where there was an equal distribution between upregulated and downregulated genes (1012 vs 1007, respectively, Fig. 8A and Table 6). Even though we had previously described that KPP muscles exhibited a similar DEG profile to human PDAC cachexia muscles, and that differed from C-26 and LLC muscles, [18], repeating an RNA-seq analysis with fresh muscle samples from KPP, C-26, and LLC mice showed that DEGs were similar this time in number between the three cachexia models (Fig. 8A and Table 6). Each model, like KPCML1 mice, expressed an approximately equal distribution of upregulated and downregulated genes.

**Table 6:** Differentially expressed genes between cachectic PDAC patients and mouse models of cancer cachexia

Human/Mouse	DEGs	Up-Regulated Genes	Down-Regulated Genes
Cachectic Patients	369	54	315
KPCML1	2019	1012	1007
KPP	3165	1810	1355
C-26	2957	1537	1438
LLC	2722	1481	1241

Given that only a small number of genes were upregulated in cachectic patients, we performed a GO biological analysis comparing the downregulated transcripts in cachectic PDAC patients with those from the other mouse models of cachexia. Consistent to our previous analysis [18], the most significant biological process in cachectic patients and KPP mice were related to the extracellular matrix (ECM) (Fig. 8B). In contrast, and similar to our previous findings [18], the most significant downregulated transcripts in muscles from the C-26 and LLC cachexia models were associated with metabolism including the production of metabolites, oxidation of organic compounds, the ribose phosphate metabolic process, and the purine metabolic process (Fig. 8B). When we compared this to muscles from adult KPCML1 mice that had metastasized, we observed that the most significant biological pathways were identical to KPP and cachectic PDAC patients, involving the ECM and extracellular structure organization. Interestingly, these leading GO pathways were indistinguishable from adult KPCML1 mice that had not metastasized (Fig. 8B). These findings highlight that KPCML1 mice, not unlike KPP, can recapitulate the cachexia phenotype of cachectic PDAC patients, and further point to the potential commonalities of mechanisms driving muscle wasting in PDAC between the primary tumor and metastatic lesions.

## Discussion

Although the prognosis of PDAC has slightly improved over the past two decades, the survival rate remains the lowest among all common tumor types [1]. Underlying causes of this poor outcome relate to the aggressive nature of this malignancy and the lack of early diagnosis, as up to 80–90% of PDAC patients initially present with locally advanced or metastatic disease [47]. A vast majority of PDAC patients, also develop cachexia, which significantly contributes to diminishing their quality of life and reducing their chance of survival [48–50]. Recent increases in patient survival from 4–12% have come in part from the use of a cocktail of chemotherapeutic agents (FOLFIRINOX) that is now routinely used to treat PDAC patients with metastatic cancer [51]. In addition, published results have shown that using FOLFIRINOX in a neoadjuvant setting for locally advanced, unresectable, or borderline resectable PDAC patients increased median progression-free survival if resection was achieved [52]. However, FOLFIRINOX is an aggressive regimen offered to select patients with a good performance status. In addition, most PDAC patients are advanced and due to their cachexia syndrome are unable to tolerate such toxic therapies. Alternative, less toxic therapies have been implemented, such as nab-paclitaxel (Abraxane) in combination with gemcitabine. However, response rates of Abraxane resulting from randomized phase III trials are less efficacious when compared to FOLFIRINOX in an independent, non-head-to-head comparison [53, 54]. Thus, it is reasonable to assume that patients currently not eligible for FOLFIRINOX could become suitable candidates for treatment if an effective anti-cachexia therapy was applied that could restore body weight and physical activity to increase the performance status.

Although multiple novel therapeutic strategies to reduce PDAC burden through immune checkpoint blockade [55–57], or combat cachexia through appetite stimulation [58], have yet to reach objective response rates or meaningful primary endpoint criteria, a series of new compounds have recently been developed that have brought renewed optimism in the fight against PDAC and its associated cachexia syndrome [52–54]. Moving forward, the success of these compounds will likely require testing in animal models that recapitulate the human phenotype as closely as possible. For effective PDAC therapy, we reason that such models would need to simulate both metastasis and cachexia. The established KPC GEMM of PDAC accurately recapitulates the genetics and pancreas pathology, as well as the progression of the disease [16]. More recently, this GEMM has also been used as a model of cachexia [19, 20]. However, as a cachexia model we found it to be limited due to its heterogeneity and stunting of growth [18]. Although KPC mice reached endpoint criteria, they often tended to be smaller, and in some cases presented with non-invasive pancreatic lesions, which is not consistent with the cachexia phenotype in patients. In addition, because the KPC GEMM initiates in utero, it is difficult to assess whether the reduction in body size is due to tissue catabolism or the inability for muscle and adipose to properly grow during neonatal development and into adulthood. To circumvent these limitations, we generated the KPP GEMM of PDAC [18]. Although we found that this GEMM appears to more closely recapitulate the cachexia seen in PDAC patients, KPP mice do not present with metastases, and therefore should not strictly be considered a model of PDAC.

To overcome some of these existing limitations in KPC and KPP GEMMs, this study was performed to optimize the orthotopic model of PDAC-induced cachexia. This model has recently gained traction in the cancer cachexia community as it allows the study of a population most at risk for cachexia; it circumvents subcutaneous transplants where often the pancreatic tumor microenvironment is not well recapitulated; the model is amendable to performing studies in immune competent mice where it maintains a correct tumor immune microenvironment which could also influence the development of cachexia; this model also overcomes the need to maintain lines of mice with complex genetics and breeding schemes; and the model allows for less time-consuming preclinical studies to be performed. An increasing number of reports have also described using variant cell lines that KPC orthotopic transplants serve as an effective model of cancer cachexia [23–26, 59, 60]. However, because metastasis is not consistently reported, it is unclear whether current experimental conditions are able to accurately recapitulate the human phenotype of PDAC.

Our findings demonstrate that transplanting KPC cells into the pancreas of immunocompetent mice can efficiently simulate both the metastatic and cachectic phenotypes of PDAC, but this depended on introducing certain optimization conditions. The first was to modify parental KPC cells and utilize instead a reconstituted metastatic line that we named KPCML1. This modification is in line with a similar reported strategy that we noted only after the completion of our study [57]. In that work, investigators transplanted 1 × 10^6^ cells into the pancreas of immunocompetent mice and achieved a rate of liver metastasis of 90%. We are unaware why such a high number of transplanted cells, which presumably would rapidly develop into a primary tumor, would give rise to such an efficient metastatic rate, when our own results showed that the efficiency of metastasis increased with decreasing numbers of implanted KPCML1 cells. We reason that this discrepancy might reflect intrinsic differences between cell lines that could account for the large differences in the number of KPC cells that we transplanted compared to what has previously been reported [21, 22]. The second modification that we made was to alter the anatomic location of implantation, choosing instead to inject tumor cells in the head of the pancreas to mimic the most common site where human PDAC develops. The third was to lower the number of implanted cells, which we found was optimal for forming both primary tumors and liver metastases, and the fourth was to perform orthotopic implantation of KPCML1 cells in adult recipient mice rather than standard young mice. Regarding the latter modification, we have not investigated why an older immunocompetent host would promote a higher rate of metastasis, but our spatial multiplex imaging data imply a connection with an increased immune tumor microenvironment. As noted above, aging can affect the tumor microenvironment to promote tumor progression [37, 38]. In addition, adult mice contain more adipose, which has been shown to promote the advancement of PDAC [61]. Therefore, it is also possible that adipokines or other adipose-related factors contributed to the higher rate of metastases in the KPCML1 model. Going forward, we will need to ensure that this phenomenon is not simply attributed to the C57Bl/6 strain that this orthotopic model is based on. Another condition that we tested that did not seem to make a difference in the rate of metastasis is the sex of recipient mice. Although new cancer statistics show that mortality rates are significantly higher in males vs females with pancreatic cancer [1], we found no evidence that rates of metastases or survival varied following the implantation of male derived KPCML1 cells in the head of the pancreas in male vs female recipient mice.

Importantly, these modified conditions described above that simulated metastasis also successfully recapitulated the cachexia phenotype. This allowed for standard outcome measures to be obtained, such as a reduction in both adipose and skeletal muscle mass. Moreover, these optimization conditions extended the median survival time of PDAC mice, which is advantageous if time points are required to study the progression of cachexia [18], or if preclinical studies are desired to test the efficacy of compounds in attenuating disease progression. Our results revealed that cachexia could be achieved in both young and adult mice, but to a slightly higher degree in the adult cohort, which also demonstrated enhanced metastasis. Significantly, KPCML1 mice exhibited the cachexia phenotype in both the metastatic and non-metastatic state, which is reflective of the patient population. Thus, we recommend that if investigators are interested in recapitulating both metastasis and cachexia phenotypes of PDAC, the KPCML1 model should be implemented in adult mice. If the primary endpoint is solely cachexia, then the model can be performed in either young or adult mice. Future studies will compare additional optimization conditions such as the combination of the implantation site and age.

In addition, GO analysis revealed that downregulated genes from muscles of PDAC patients with cachexia and those from KPCML1 mice exhibited similar pathways, providing molecular evidence that the KPCML1 model recapitulates the phenotype of human PDAC. We also determined that these same pathways, characteristic of an altered ECM, were observed in muscles from both KPCML1 non-metastatic and metastatic mice. This suggests that driver mechanisms leading to muscle wasting derive primarily from the primary tumor, and potentially, that future anti-cachexia therapeutics could be designed with the goal of treating cachexia in both early and advanced stages of cancer.

Furthermore, we discovered in this study that the optimization of the orthotopic model did not make a difference in distinguishing this model over other mouse cancer cachexia models with regards to the C18:C24 ceramide ratio. Analogous to PDAC cachexia patients, circulating levels of C18:C24 were elevated at endpoint in KPCML1 mice. However, this increase was also observed in the serum from the parental KPC cell orthotopic model, KPP, LLC, and to a large degree C-26 tumor mice. Therefore, if the C18:C24 ceramide ratio is proven in subsequent studies to become a *bona fide* biomarker of cancer cachexia, it will likely be reflective in multiple cancer cachexia animal models.

Although our study describes an optimized orthotopic mouse model that recapitulates the metastatic and cachectic features of PDAC, the model itself is not without its own limitations. Comparative analysis of the pancreas histology reveals that the orthotopic model is not as accurate as GEMMs in reflecting the human phenotype of the tumor microenvironment, which exhibits a desmoplastic stroma and a complex interplay between tumor and multiple subpopulations of immune and fibroblast cells. We recognize such a condition, as well as others, will need to be tested in subsequent studies to continue improving the optimization of this model of PDAC cachexia. Doing so will be important for enhancing our understanding of the underlying mechanisms and treatment strategies of PDAC and the resulting cachexia syndrome. In addition, because feeding was not controlled, the possibility exists that similarities in transcriptomes between metastatic and non-metastatic mice may be linked to a decrease in food intake as KPC mice approach their endpoint.

## Conclusions

We were able to successfully optimize a KPC orthotopic model of PDAC to more closely recapitulate the phenotypes of patients that commonly present with liver metastasis and cachexia. This model also revealed that similar to patients with PDAC, KPC mice with PDAC exhibit cachexia in both an early and advanced stage of the disease, and that the mechanisms driving skeletal muscle wasting in cachexia might also be similar between the early and advanced stages of PDAC. Moreover, we find that elevation of the C18:C24 ceramide ratio in the circulation of KPC PDAC mice matches that of other cancer cachexia mouse models and compares to what we have recently reported in plasma from cachectic PDAC patients [46], supporting further exploration of C18:C24 as a potential biomarker of cancer cachexia. Together, our optimized orthotopic model is likely to advance our understanding of the mechanisms of PDAC cachexia, as well as to facilitate the evaluation of future anti-PDAC and anti-cachexia therapeutics.

### Supplementary Information

**Supplementary Material 1. **

## Data Availability

The raw RNA-Seq data set has been submitted to the NCBI Gene Expression Omnibus (GEO) and will be publicly accessible upon publication.

## Abbreviations

AW: Ascites weight
BlW: Blood withdrawn
BRACA 1/2: BReast CAncer gene 1/2
BW: Body weight
C-26: Colon-26
DSP: Digital Spatial Profiling
ECM: Extracellular matrix
GAST: Gastrocnemius
GEMM: Genetically Engineered Mouse Model
Has1: Hyaluronan Synthase 1
Klk8: Kallikrein-8
KPC: Kras + /LSL-G12D, Trp53 + /R270H, Pdx1 + /Cre
KPCML1: KPC Metastasis Large Nodule 1
KRAS: Kirsten rat sarcoma viral oncogene homolog
KPP: Kras + /G12D, Ptf1a + /ER-Cre, Ptenf/f
LLC: Lewis Lung Carcinoma
PanIN: Pancreatic Intraepithelial Neoplasia
PDAC: Pancreatic ductal adenocarcinoma
QUAD: Quadriceps
SMAD4: Mothers against decapentaplegic homolog 4
Syt8: Synaptotogmin 8
TA: Tibialis anterior
TMBW: Total measured body weight
TP53: Tumor protein p53
TW: Tumor weight
