# Supplementary material for: Optimization of a mouse model of pancreatic cancer to simulate the human phenotypes of metastasis and cachexia
Source: BMC Cancer. 2024 Apr 4;24:414. doi: 10.1186/s12885-024-12104-0 (PMC10993462; doi:10.1186/s12885-024-12104-0)
Supplement: Supplementary file 1 — Supplementary Material 1. [file 12885_2024_12104_MOESM1_ESM.pdf]

Supplemental Figure 1

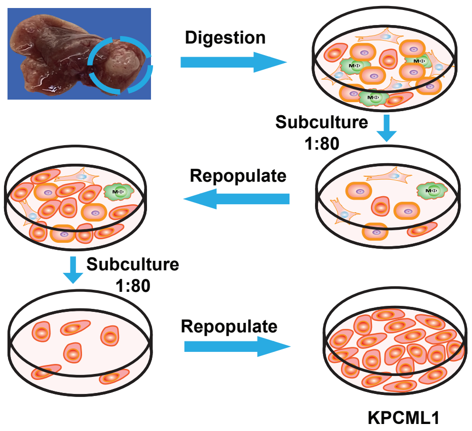

Supplemental Figure 2

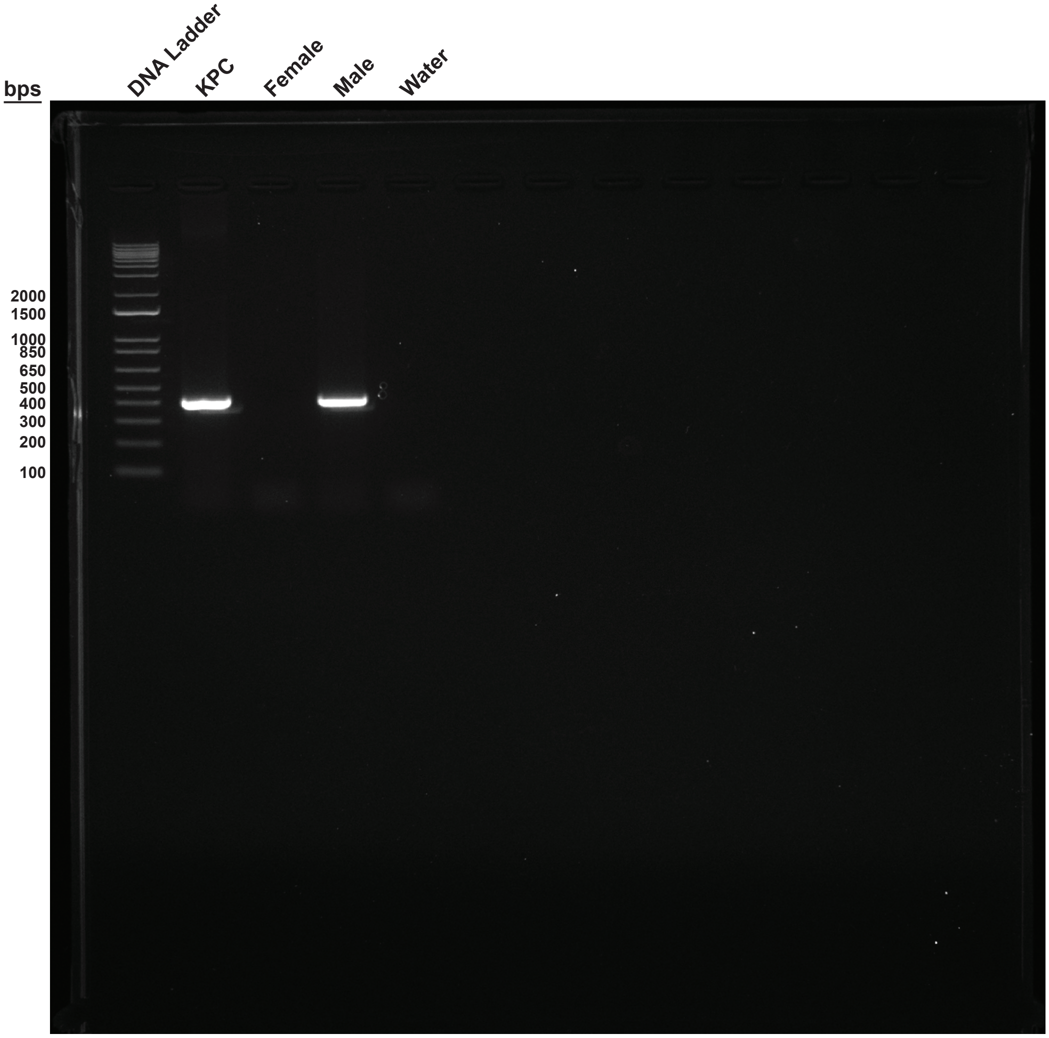

Supplemental Figure 3

A

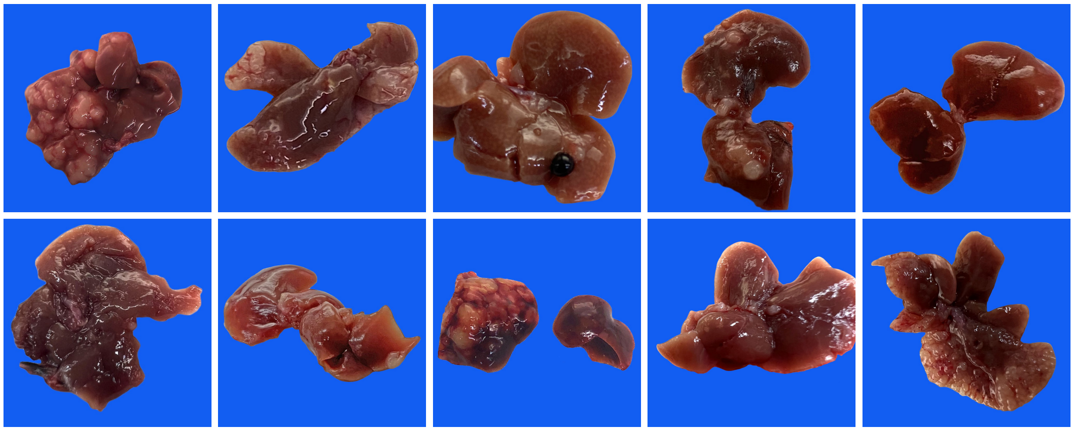

B

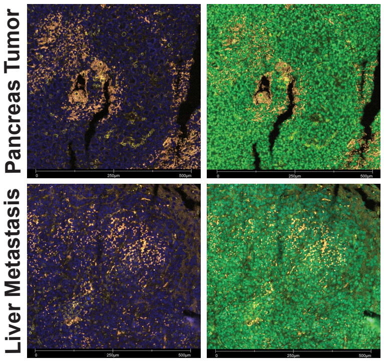

C

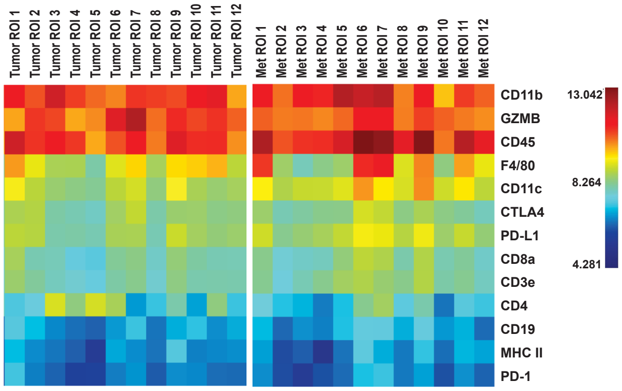

Supplemental Figure 4

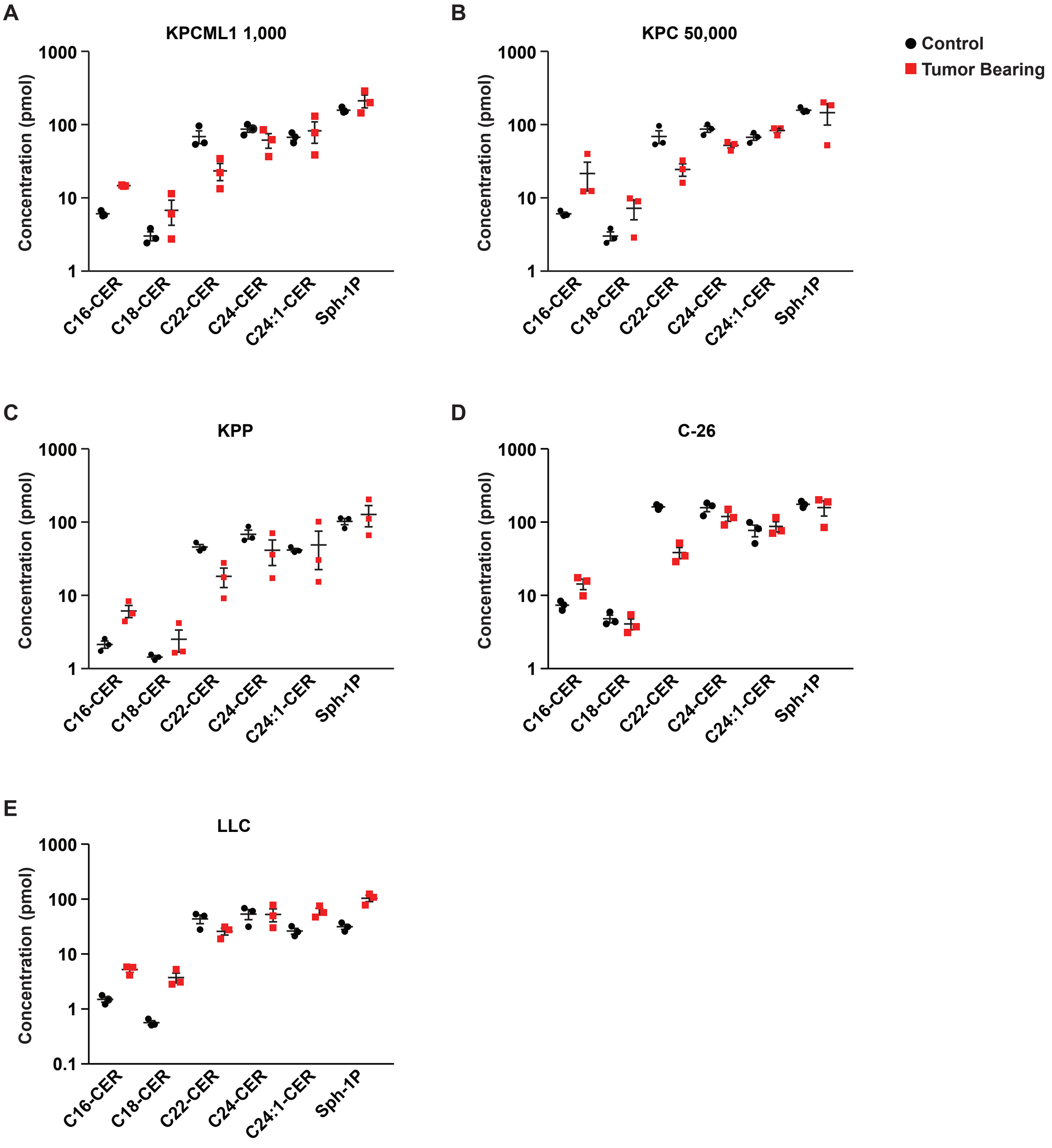

Supplemental Figure 5

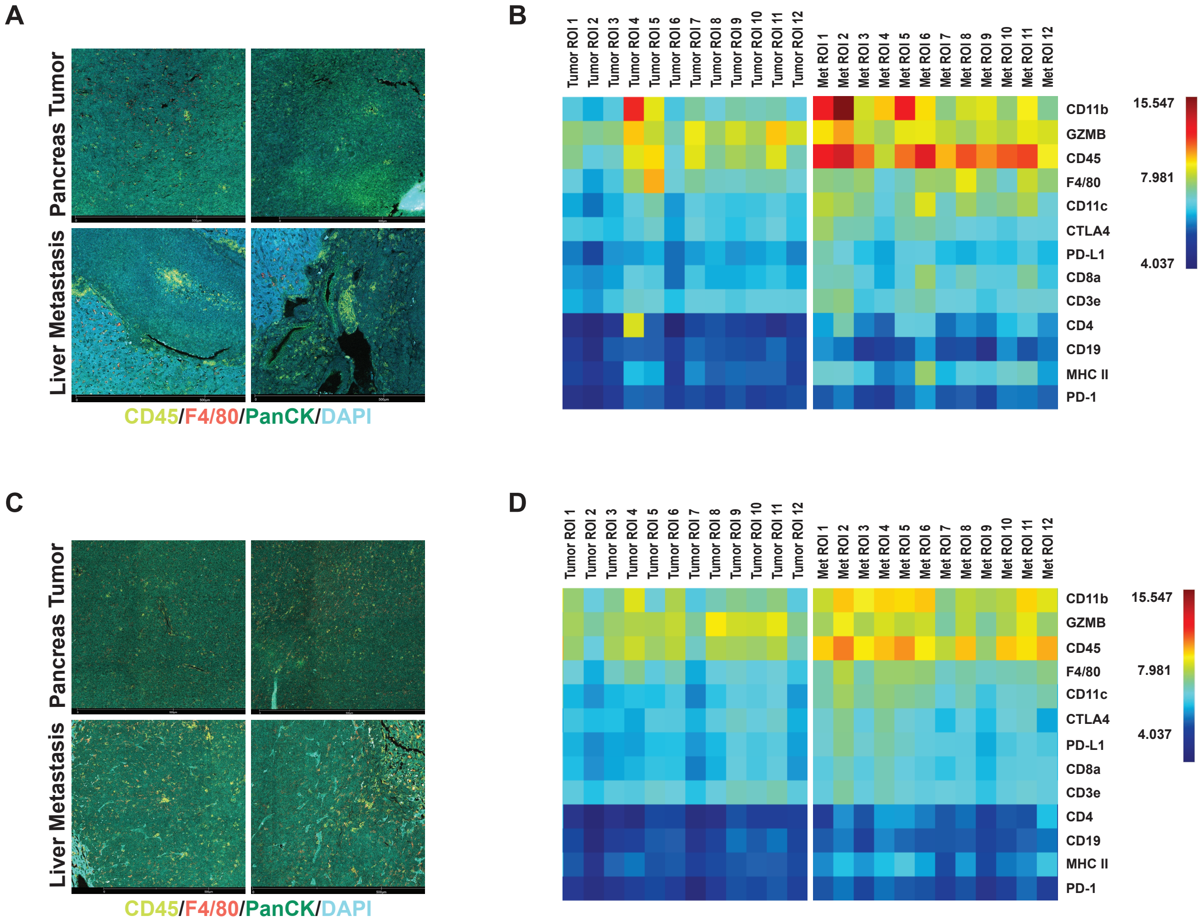

SUPPLEMENTAL FIGURE LEGENDS

Supplemental Figure 1: *Establishment of the KPCML1 cell line*. Schematic representation of how the KPCML1 cell line was generated. The illustration depicts a metastatic liver nodule that was removed, digested, and subcultured and repopulated 3 times to generate a purified epithelial tumor cell line, which was named KPCML1.

Supplemental Figure 2. *KPC cells were derived from male mice.* Agarose gel image of PCR products for amplification of murine male specific gene *sry* from genomic DNA extracted from KPC cell line as well as from tails of either male or female mice.

Supplemental Figure 3. *KPCML1 mice exhibit high rates of liver metastases with similar tumor immune microenvironments.* (A) Representative photographs of livers containing single or multiple metastatic nodules that were removed from mice (n=10) each injected with KPCML1 cells. (B) Images representing digital spatial profiling with an immune-oncology panel comparing the primary and metastatic KPCML1 tumors. (C) A cluster of immune markers were identified from selected ROIs and heat map profiles were generated and compared between the primary and metastatic tumor.

Supplemental Figure 4. *Plasma sphingolipid content is altered in serum from KPCML1 mice and other mouse models of cancer cachexia*. A panel of serum ceramide levels of, C16, C18, C22, C24, C24:1, and S1P were measured in KPCML1 mice (A), the KPC orthotopic model (B), as well as in the KPP (C), C-26 (D), and LLC (E) mouse models of cancer cachexia.

Supplemental Figure 5. *Metastatic lesions from adult KPCML1 mice exhibit an enhanced immune tumor microenvironment.* (A, C) Images representing digital spatial profiling with an immune-oncology panel comparing the primary and metastatic KPCML1 tumors in adult mice, n=2. (B,D) A cluster of immune markers were identified from selected ROIs and heat map profiles were generated and compared between the primary and metastatic tumor, n=2.

Supplemental Table 1. Tumor weights and survival from orthotopic implantation of KPC cell lines

Sex	# of Mice	Cell Line	Cell #	# of Mice with Tumors	% Tumors	Tumor Weight (g)	Median Survival (days)
Orthotopic Implantations into the Tail of the Pancreas							
Female	7	KPC	50,000	7	100	5.32 ± 0.79	ND
Female	6	KPC 2838c3	50,000	6	100	ND	ND
Female	6	KPC 6419c5	50,000	6	100	ND	ND
Orthotopic Implantations into the Head of the Pancreas							
Female	7	KPC	10,000	6	85.7	ND	51
Female	5	KPC 2838c3	10,000	5	100	ND	51
Female	5	KPC 6419c5	10,000	5	100	ND	35
Orthotopic Implantations into the Head of the Pancreas							
Male	6	KPC	5,000	6	100	ND	53
Male	6	KPC	10,000	6	100	ND	51
Male	5	KPC	20,000	5	100	ND	46
Orthotopic Implantations into the Head of the Pancreas							
Male	5	Matrigel	0	0	0	0	ND
Male	6	KPCML1	500	3	50	2.11 ± 2.79	47
Male	5	KPCML1	1,000	5	100	6.22 ± 2.44	53
Male	6	KPCML1	10,000	6	100	ND	37
Orthotopic Implantations into the Head of the Pancreas							
Male	10	KPCML1	1,000	10	100	7.07 ± 3.16	44
Female	10	KPCML1	1,000	10	100	6.41 ± 1.62	47

Supplemental Table 2. Tumor weights and survival from orthotopic implantation of KPCML1 cells in young and adult mice

Sex	Age	# of Mice	Cell Line	Cell #	# of Mice with Tumors	% of Tumors	Tumor Weight (g)	Median Survival (days)
Male	Young	10	KPCML1	0	10	100	5.64 ± 3.79	46
Male	Adult	10	KPCML1	0	10	100	6.08 ± 3.70	36
